# Theoretical Study of Alkaline-Earth Metal (Be, Mg, and Ca)-Substituted Aluminum Nitride Nanocages With High Stability and Large Nonlinear Optical Responses

**DOI:** 10.3389/fchem.2022.918704

**Published:** 2022-06-21

**Authors:** Hui-Min He, Hui Yang, Ying Li, Zhi-Ru Li

**Affiliations:** ^1^ Department of Physics, Institute of Computational and Applied Physics, Taiyuan Normal University, Jinzhong, China; ^2^ School of Chemistry and Chemical Engineering, Shanxi Datong University, Datong, China; ^3^ Laboratory of Theoretical and Computational Chemistry, Jilin University, Changchun, China

**Keywords:** nonlinear optical response (NLO), excess electron compound, aluminum nitride nanocage, first hyperpolarizabilities, density functional theory

## Abstract

By replacing one Al or N atom of aluminum nitride nanocage Al_12_N_12_ with an alkaline-earth metal atom, two series of compounds, namely, M@Al_12_N_11_ and M@Al_11_N_12_ (M = Be, Mg, and Ca), were constructed and investigated in theory. The substituted effect of alkaline-earth metal on the geometric structure and electronic properties of Al_12_N_12_ is studied in detail by density functional theory (DFT) methods. The calculated binding energies, HOMO–LUMO gaps, and VIE values of these compounds reveal that they possess high stability, though the NBO and HOMO analyses show that they are also excess electron compounds. Due to the existence of diffuse excess electrons, these alkaline-earth metal-substituted compounds exhibit larger first hyperpolarizabilities (*β*_0_) than pure Al_12_N_12_ nanocage. In particular, these considered compounds exhibit satisfactory infrared (IR) (>1800 nm) and ultraviolet (UV) (˂ 250 nm) transparency. Therefore, these proposed excess electron compounds with high stability may be regarded as potential candidates for new UV and IR NLO molecules.

## Introduction

Over the past few decades, the design and synthesis of nonlinear optical (NLO) materials with excellent performance have exerted a tremendous fascination on researchers in consideration of their widespread applications in optics and optoelectronics (Marder Seth et al., 1994; Priyadarshy et al., 1996; Nakano et al., 2002; Zhang et al., 2005; Xiao et al., 2008; Xu et al., 2013). Up to now, abundant strategies have been proposed to acquire high-performance NLO materials of the new type, for instance, designing donor–π-conjugated-bridge-acceptor (D–π-A) models (Kanis et al., 1994), decorating or modifying sp^2^-hybridized carbon nanomaterials (Bai et al., 2013; Muhammad et al., 2013; Yu et al., 2013; Karamanis et al., 2014; Zhou et al., 2014), synthesizing octupolar molecules (Ja Lee et al., 2003), utilizing a multideck sandwich cluster as the building block (Wang et al., 2015), applying the bond length alternation (BLA) theory (Meyers et al., 1994), enhancing push–pull effects (Liu et al., 2010), and doping metal atoms (Di Bella, 2001; Zhong et al., 2012b; Wu et al., 2014), etc. In particular, Chen et al. (2004) and Li et al. (2004) have revealed that the introduction of loosely bound excess electrons into a molecule is an effective measure to dramatically enhance its NLO response. Therein, the diffuse excess electron is responsible for improved NLO response. Subsequently, a lot of compounds with dispersed excess electrons, namely, excess electron compounds, are designed in theory, and as expected, they exhibit considerable large NLO responses (Zhong et al., 2015).

In previous studies, it has been found that alkali-metal-doped organic complexants exhibit large first hyperpolarizabilities (Chen et al., 2005; Xu et al., 2007; Wang et al., 2012). In these systems, excess electrons are formed when organic complexants push/pull valence electrons of alkali metal atoms. Therefore, such systems were classified as excess electron compounds, where the alkali metal atom acts as the excess electron source. However, the introduction of active alkali metal atoms reduces stability of these compounds. Therefore, selecting the proper excess electron source to achieve new excess electron compounds will be an efficient way to obtain NLO materials with high stability. To achieve this aim, we have used alkaline-earth metal atoms as an electron source to design several types of excess electron compounds (He et al., 2017a; He et al., 2017b; He et al., 2019) with large first hyperpolarizabilities and satisfying stability.

On the other word, fullerene-like hollow nanocages with noncarbon have attracted great attention owing to their special optoelectronic properties in recent years (Golberg et al., 1998; Strout, 2000; Fu et al., 2001; Stafström et al., 2001; Bertolus et al., 2004; Wang et al., 2005; Beheshtian et al., 2011; Ahmadi Peyghan et al., 2012; Beheshtian et al., 2012c). In particular, Group III–V nitrides are the most promising nanoscale materials in various fields. Therefore, a lot of theoretical and experimental studies have been carried out on the Group III-V fullerene-like nanostructures, especially the most significant AlN nanocages because of their high thermal conductivity and chemical stability (Silaghi-Dumitrescu et al., 1996; Oku et al., 2004; Koi et al., 2005; Yang et al., 2005; Zhang and Zhang, 2005; Zope and Dunlap, 2005; Li et al., 2007; Beheshtian et al., 2012a; Liu et al., 2013; Saeedi et al., 2013). *Ab initio* calculations revealed that the Al_12_N_12_ nanocage is the most stable AlN nanostructure and thereby can be regarded as an ideal inorganic fullerene-like candidate (Wu et al., 2003). Considering the unique structural and electronic structure of this cage, it has been used as an excellent starting material to design NLO molecules. For instance, two inorganic electrides, M@Al_12_N_12_(M = Li, Na, and K) (Niu et al., 2014) and M_3_O@Al_12_N_12_ (M = Li, Na, and K) (Sun et al., 2016), were achieved by doping alkali metal atoms and superalkali clusters on the Al_12_N_12_ nanocage, while the M@Al_12_N_11_ and M@Al_11_N_12_ compounds were designed by substituting one atom of Al_12_N_12_ with an alkali-metal atom (Maria et al., 2016). All these Al_12_N_12_-based excess electron compounds exhibit considerably high NLO responses.

In order to enhance the NLO responses and stability of such Al_12_N_12_-based compounds, two series of inorganic compounds, M@Al_12_N_11_ and M@Al_11_N_12_ (M = Be, Mg, and Ca), were obtained by substituting Al or N atom in the Al_12_N_12_ with alkaline-earth metal in the current work. We mainly focus on the following issues: 1) Can loosely bound excess electrons that can dramatically enhance molecular NLO response be generated in these proposed inorganic compounds with alkaline-earth substituted? 2) Do these novel compounds possess larger stability and first hyperpolarizability (*β*_0_) than those of previously reported alkali-metal-substituted systems M@Al_12_N_11_ and M@Al_11_N_12_ (M = Li, Na, and K)? Our results revealed that these inorganic compounds M@Al_12_N_11_ and M@Al_11_N_12_ (M = Be, Mg, and Ca) indeed contain diffuse excess electrons and thus exhibit larger *β*_0_ values than alkali-metal-substituted systems. In particular, These excess electron compounds exhibit excellent infrared (IR) (>1800 nm) and ultraviolet (UV) (˂ 250 nm) transparency for their potential applications with modern laser frequency conversion technology and optical parameter oscillator processes (Zhang et al., 2009; Luo et al., 2014).

## Computational Details

The geometric structures with all real frequencies are obtained by using a combination of Becke’s hybrid 3-parameter exchange functional (Becke, 1993a) and Lee–Yang–Parr’s correlation function (Lee et al., 1988) (B3LYP). In a previous study, the Pople-type (Bilbrey et al., 2013) basis set 6-31+G(d) was selected because it has been proved to be reliable for the geometry optimization of similar systems (Niu et al., 2014). Natural bond orbital (NBO) analyses, vertical ionization energies (VIE), and binding energy (*E*_b_) calculations were also performed at the B3LYP/6-31+G(d) level. The binding energy of the alkaline-earth metal atom M was calculated using the counterpoise procedure (Alkorta and Elguero, 1999) and is defined as follows: For M@Al_12_N_11_,

$$E_{\text{b}}=\left(E_{{\text{Al}}_{12}{\text{N}}_{11}}\;+\;E_{\text{M}}\right)-E_{\text{M}@{\text{Al}}_{12}{\text{N}}_{11.}}$$ (1)

For M@Al_12_N_11_,

$$\;\;\;\;\;\;\;E_{\text{b}}=\left(E_{{\text{Al}}_{11}{\text{N}}_{12}}\;+\;E_{\text{M}}\right)-E_{\text{M}@{\text{Al}}_{11}{\text{N}}_{12.}}$$ (2)

The VIE is the energy difference between neutral molecule and cation systems at the neutral optimization geometry (Wang et al., 2012; Bai et al., 2013; Sun et al., 2015; He et al., 2019).

Polarizability and first hyperpolarizability calculations are carried out on long-range correlated methods CAM-B3LYP (Tawada et al., 2004; Yanai et al., 2004) and BHandHLYP (Becke, 1993b) in conjunction with the 6-31+G(d) basis set. As all the systems are in the doublet states, values of spin eigenvalue ⟨S^2^⟩ involved in the structural optimization and NLO computations are in the range of 0.760–0.789, which shows an error range of 1.3%–4.9%, indicating that the spin contamination is negligible in the current calculations.

The static polarizability (*α*_0_) and the first hyperpolarizability (*β*_0_) are defined as follows:

$$\alpha_{0}=\frac{1}{3}\left(a_{xx}+a_{yy}+a_{zz}\right),$$ (3)

$$\beta_{0}=\sqrt{\beta_{x}^{2}+\beta_{y}^{2}+\beta_{z}^{2}},$$ (4)

where $\beta_{i}=\frac{3}{5}\left(\beta_{iii}+\beta_{ijj}+\beta_{ikk}\right),\;i,j,k=x,y,z.$

For electronic transition properties, the transition energy Δ*E*, oscillator strength *f*_0_, and the difference of dipole moment Δ*μ* between the ground and the crucial excited state are estimated by the time-dependent density functional theory, TD-CAM-B3LYP, with the 6-31+G(d) basis set. Simultaneously, ultraviolet-visible–infrared (UV-VIS-IR) absorption spectra of all the systems were also obtained at the same level. All the UV-VIS-IR spectra were reflected with Gaussian curves under a full-width at half-maximum (FWHM) of 0.10 eV.

All of the calculations were carried out by using the Gaussian 16 program package (Frisch et al., 2016). Molecular configurations and molecular orbital (MO) plots were generated with the GaussView program (Dennington et al., 2016).

## Results and Discussion

**FIGURE 1:**
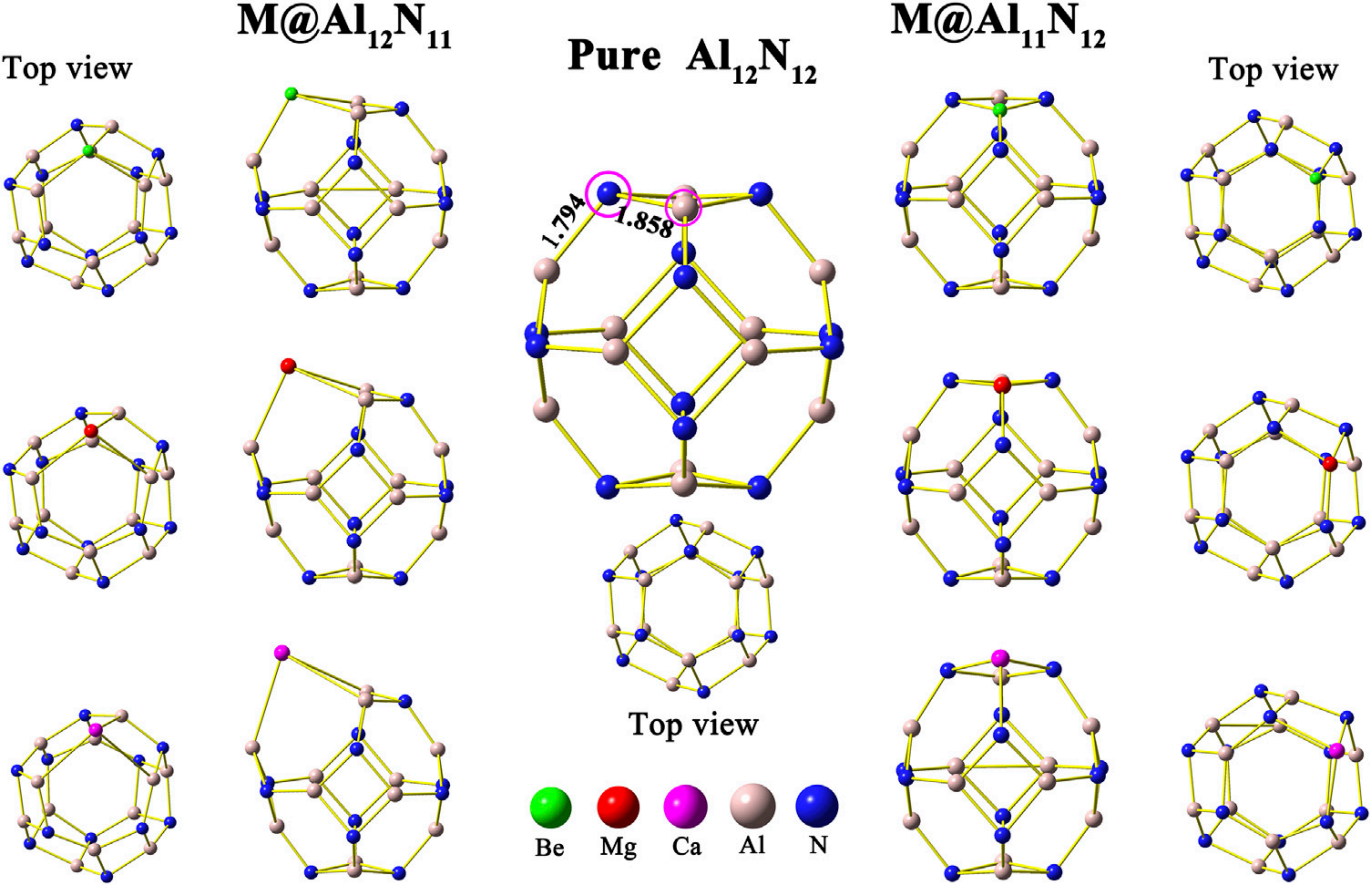
Optimized geometric structure of undoped Al_12_N_12_ and alkaline-earth metal-doped M@Al_12_N_11_ and M@Al_11_N_12_ (M = Be, Mg, and Ca).

First, the isolated aluminum nitride nanocage Al_12_N_12_ was optimized, and its structure is shown in Figure 1. In this study, the pure Al_12_N_12_ nanocage is found to be a *T*_h_-symmetric fullerene-like cage consisting of six 4-membered rings and eight 6-membered rings, in which the Al–N bond lengths are 1.794 and 1.858 Å, respectively, in consistent with earlier reports (Beheshtian et al., 2012a; Beheshtian et al., 2012b; Niu et al., 2014). Then, the initial geometry structures of M@Al_12_N_11_ and M@Al_11_N_12_ (M = Be, Mg, and Ca) were constructed by replacing one atom (Al or N) in the Al_12_N_12_ nanocage with an alkaline-earth metal atom M. In the M@Al_12_N_11_ series, one alkaline-earth metal atom is substituted for one nitrogen atom, whereas one aluminum atom is replaced with one alkaline-earth metal atom in M@Al_11_N_12_. After optimization, six equilibrium conformations of M@Al_12_N_11_ and M@Al_11_N_12_ have been obtained and are shown in Figure 1. The selected geometrical parameters, VIE values, and HOMO–LUMO gap values of these resulting compounds as well as the binding energies and NBO charges of alkaline-earth metal atoms in them are listed in Table 1.

**TABLE 1:** Symmetry, average M–Al bond length (in Å), average M–N bond length (in Å), HOMO–LUMO gap (H-L Gap, in eV), and vertical ionization energies (VIE, in eV) as well as the binding energy (*E*_b_, in kcal/mol) and NBO charge of alkaline-earth metal atom for M@Al_12_N_11_ and M@Al_11_N_12_ (M = Be, Mg, and Ca).

Property	Al_12_N_12_	M@Al_12_N_11_			M@Al_11_N_12_		
		Be	Mg	Ca	Be	Mg	Ca
Symmetry	*T*_h_	*C*_s_	*C*_s_	*C*_1_	*C*_s_	*C*_s_	*C*_1_
Bond length	1.794/1.858	2.374	2.787	3.206	1.685	2.051	2.385
*q* ^*M*^		-0.358	0.394	0.938	1.101	1.591	1.798
VIE	19.75	6.66	6.22	5.93	8.95	8.80	7.78
*E*_b_		62	33	37	244	163	173
H-L gap	3.84	2.32	2.11	1.73	1.19	1.22	1.19

As shown in Table 1, when one nitrogen or aluminum atom is replaced, the original Al-N bond is elongated. That is to say, the bond lengths of M–Al and M–N in the displaced systems are longer than those of the original Al-N in the pure Al_12_N_12_ nanocage. Nevertheless, the Be-N bond is a special case in Be@Al_11_N_12_. The Be-N bond in the Be@Al_11_N_12_ decreases by ca. 0.175 Å as compared with the Al-N bond in the isolated Al_12_N_12_ nanocage, which may be attributed to the fact that the nitrogen atom possesses larger electronegativity and the Be atom has smaller atomic radii. It is also found that the bond lengths of M–Al and M–N show a monotonous increase with the increasing atomic number of M. In addition, the M–Al bond length of M@Al_12_N_11_ series exhibits a larger increment than the M-N bond length of the M@Al_11_N_12_ series as compared with the Al-N bond length of pure Al_12_N_12_ nanocage.

**FIGURE 2:**
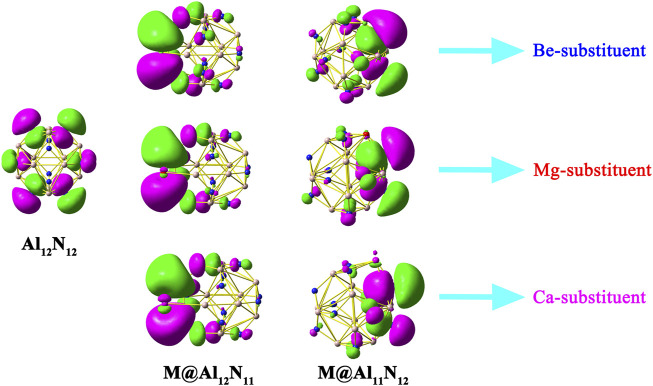
HOMOs of Al_12_N_12_, M@Al_12_N_11_, and M@Al_11_N_12_ (M = Be, Mg, and Ca) systems.

Figure 2 displays HOMOs of Al_12_N_12_ nanocage, M@Al_12_N_11_, and M@Al_11_N_12_ (M = Be, Mg, and Ca). It can be seen that the HOMO of a pure Al_12_N_12_ nanocage consists of p atomic orbitals of N atoms. Differently, all the HOMOs of M@Al_12_N_11_ and M@Al_11_N_12_ systems possess diffuse excess electrons, reflecting the unique electric characteristics of these studied compounds. Therefore, these proposed M@Al_12_N_11_ and M@Al_11_N_12_ (M = Be, Mg, and Ca) systems can be regarded as a new type of inorganic excess electron compounds. Interestingly, it is found that these systems exhibit almost the same HOMOs.

NBO analysis was carried out to analyze charge transfer in nanocages and electronic structures of these systems. The computed NBO charges on alkaline-earth metal atoms in these studied compounds are given in Table 1. From Table 1, it can be seen that the NBO charges of alkaline-earth metal atoms in M@Al_11_N_12_ (M = Be, Mg, and Ca) are in the range of (1.101–1.798) |e|, which is much larger than the charges (0.496–0.788) |e| (Maria et al., 2016) on alkali metal atoms in M@Al_11_N_12_ (M = Li, Na, and K) and the charges of (0.32-0.60) |e| (Ullah et al., 2020) on alkaline-earth metal atoms in AEM@Al_12_N_12_ (M = Be, Mg, and Ca), indicating that more charges are transferred from alkaline-earth metal atoms to the nanocages in these compounds. In addition, it is also found that the charges on M atoms in the M@Al_11_N_12_ series are more electropositive than those of the M@Al_12_N_11_ series, which may be attributed to the fact that the electronegativity of the nitrogen atom is larger than that of the aluminum atom. When an alkaline-earth metal atom is substituted for an Al atom or N atom, the nitrogen atom has a stronger ability to absorb electrons than the Al atom. Therefore, introducing M atoms in the M@Al_11_N_12_ series loses more electrons and displays more electropositivity. Additionally, it is found that the charges on M atoms increase along with the increasing M atomic number in both series because of the increasing electropositivity of M. Moreover, the NBO analysis also reveals that the alkaline-earth metal atoms can serve as the source of diffuse excess electrons for these excess electron compounds.

**FIGURE 3:**
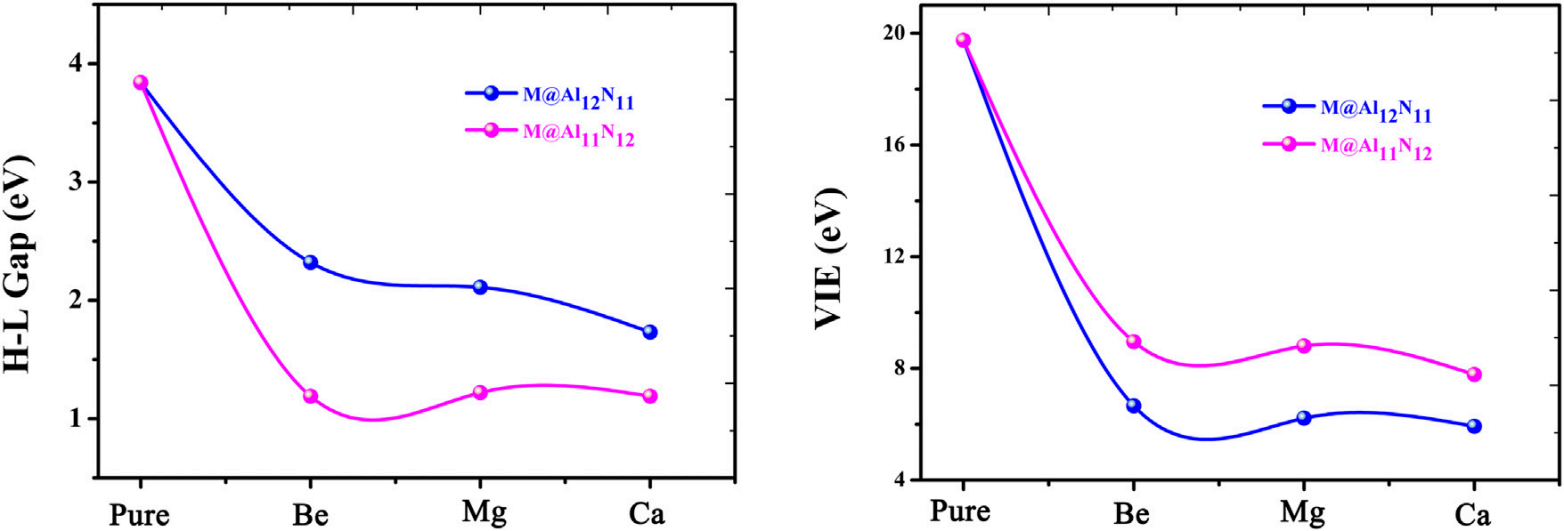
Relationship between H-L Gaps and VIE values with the atomic number of alkaline-earth metals.

It is known that the stability of molecules is of great significance for their synthesis in the laboratory and further application in practice. Moreover, the kinetic stability, chemical reactivity, and optical polarizability of any molecule can be described from the energy gap between the highest occupied molecular orbital (HOMO) and the lowest unoccupied molecular orbital (LUMO). HOMO–LUMO gaps of all the studied excess electron compounds are calculated and are summarized in Table 1. As can be seen from Table 1, pure Al_12_N_12_ has a large band gap of 3.84 eV, which is a barrier in the way of its applications in electronic devices. As compared with Al_12_N_12_, a crucial decrease in the HOMO–LUMO gap was noticed for all replaced systems. That is to say, the HOMO–LUMO gaps of M@Al_12_N_11_ and M@Al_11_N_12_ are reduced to (1.19–2.32) eV. Even so, the gap values of M@Al_12_N_11_ (M = Be, Mg, and Ca) compounds are much larger than those (1.39–1.78) eV (Maria et al., 2016) of M@Al_12_N_11_ (M = Li, Na, and K) compounds, comparable to that (1.57 eV) (Wang et al., 1999) of the kinetically stable C_60_, and those (1.59–3.79) eV (Ullah et al., 2020) of AEM@Al_12_N_12_ (M = Be, Mg, and Ca), suggesting the large chemical stability of the studied M@Al_12_N_11_ (M = Be, Mg, and Ca) species. From Figure 3, it is also found that the gap values decrease along with the decreasing M atomic number for the M@Al_12_N_11_ series, whereas they are hardly equal for the M@Al_11_N_12_ series.

Furthermore, the large electronic stability of these excess electron compounds can also be characterized by their higher vertical ionized energies (VIEs) of (5.93–8.95) eV, which are slightly higher than the reported values of inorganic and organic excess electron compounds (Chen et al., 2005; Zhong et al., 2012a; He et al., 2017a; He et al., 2017b; Ullah et al., 2020), indicating that these proposed nanocage compounds exhibit high electronic stability. Figure 3 demonstrates the alkaline-earth metal atomic number effect on VIE values, namely, the larger the atomic number, the smaller the VIE value.

Additionally, the binding energies (*E*_b_) of these designed M@Al_12_N_11_ and M@Al_11_N_12_ compounds are also calculated and given in Table 1. The *E*_b_ values are defined as the negative of the intramolecular interaction energies between the alkaline-earth metal M and the remaining Al_12_N_11_ or Al_11_N_12_ moieties. Thus, the larger the *E*_b_ value is, the stronger the interaction between two subunits. From Table 1, it can be seen that all the proposed excess electron compounds exhibit much larger *E*_b_ values of (33.0-244.0) kcal/mol than those (31-89 kcal/mol) (Maria et al., 2016) of previously reported alkali-metal substituted compounds, indicating that alkaline-earth metal atoms are more tightly bound to the remaining Al_12_N_11_ or Al_11_N_12_ units than the alkali metal atoms in M@Al_12_N_11_ and M@Al_11_N_12_ (M = Li, Na, and K). More importantly, the M@Al_11_N_12_ (M = Be, Mg, and Ca) series exhibit extremely large *E*_b_ values up to (173-244) kcal/mol, which are far more than those of (64-89) kcal/mol for M@Al_11_N_12_ (M = Li, Na, and K) (Maria et al., 2016). Thus, as compared with alkali metal atoms, the introduction of alkaline-earth metal atoms into the Al_12_N_12_ nanocage can produce more stable species with excess electrons. In addition, the *E*_b_ values of proposed alkaline-earth metal substituted excess electron compounds are also comparable to those (59-106) kcal/mol of small clusters with alkaline-earth metal atoms (Srivastava et al., 2018).

**FIGURE 4:**
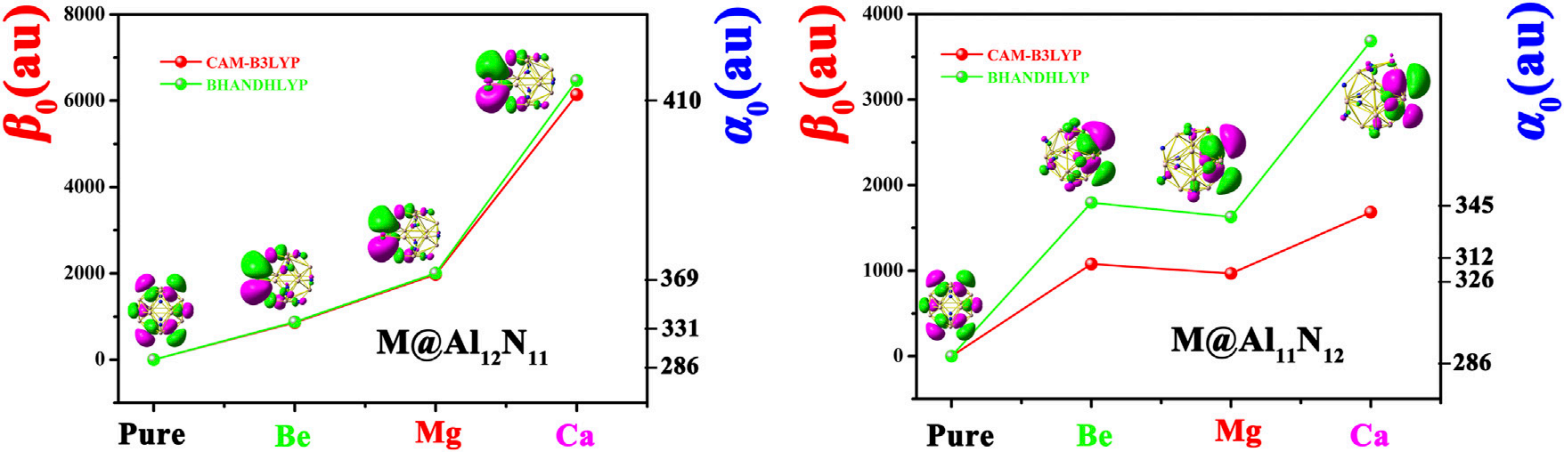
Dependences of *α*_0_ and *β*_0_ values on the M atomic number for Al_12_N_12_, M@Al_12_N_11_, and M@Al_11_N_12_ and the first hyperpolarizability comparison of two different methods.

To evaluate nonlinear optical response, the dipole moments (*µ*_0_), polarizabilities (*α*_0_), and first hyperpolarizabilities (*β*_0_) of pure Al_12_N_12_ and the proposed M@Al_12_N_11_ and M@Al_11_N_12_ compounds were calculated and are summarized in Table 2. To better visualize the results, the dependences of the polarizability (*α*_0_) and first hyperpolarizability (*β*_0_) values on the alkaline-earth metal atomic number are exhibited in Figure 4. Our results revealed that the *α*_0_ (286 au) of the Al_12_N_12_ nanocage is increased to 319-410 au for replaced compounds, indicating that the substitution of alkaline-earth metal atoms virtually affects the *α*_0_ value of the Al_12_N_12_ molecule. As shown in Table 2 and Figure 4, the *α*_0_ changes in the order of 331 au (Be) < 369 au (Mg) < 410 au (Ca) in the M@Al_12_N_11_ series and similarly, varies in the order of 312 au (Be) < 326au (Mg) < 345au (Ca) in the M@Al_11_N_12_ series. In short, *α*_0_ increases along with the increasing M atomic number. Also, it is observed that the M@Al_12_N_11_ exhibits a relatively larger *α*_0_ value than the corresponding M@Al_11_N_12_, indicating that the excess electrons in the HOMOs of the former are more diffuse than the latter because static polarizability is sensitive to electronic delocalization.

**TABLE 2:** Static polarizability (*α*_*0*_), static first hyperpolarizability *β*_*0*_, oscillator strength (*f*_o_), transition energy (*∆E*, in eV), and difference of dipole moment (*∆μ* in D) between the ground and the crucial excited states and crucial transition for M@Al_12_N_11_ and M@Al_11_N_12_ (M = Be, Mg and Ca).

		*α*_*0*_	*β*_*0*_ ^a^	*β*_*0*_ ^b^	*f*_o_	*∆E*	*∆μ*	Crucial transition
Al_12_N_12_	-	286	0	0	0.0176	4.759	0	-
M@Al_12_N_11_	Be	331	861	875	0.0434	3.32	2.53	*α*H→*α*L+3 (23.8%)
	-							*α*H→*α*L+5 (19.1%)
	-							*β*H-1→*β*L (17.2%)
	Mg	369	1979	2006	0.1034	2.93	2.27	*α*H-1→*α*L+1 (37.1%)
	-							*β*H→*β*L+2 (41.2%)
	Ca	410	6,140	6,473	0.0783	2.83	2.19	*α*H-1→*α*L+2 (18.9%)
	-							*α*H-1→*α*L+1 (15.7%)
	-							*β*H→*β*L+2 (39.7%)
M@Al_11_N_12_	Be	312	1,079	1796	0.0181	0.77	3.40	*β*H→*β*L (87.1%)
	Mg	326	969	1,628	0.0354	0.97	2.53	*β*H→*β*L (64.5%)
	-							*β*H-2→*β*L (19%)
	Ca	345	1,683	3,687	0.0420	0.92	2.09	*β*H→*β*L (49.6%)
	-							*β*H-1→*β*L (32.7%)

a for CAM-B3LYP level.

b for BHandHLYP level.

Due to the pure Al_12_N_12_ nanocage being centrosymmetric, its *β*_0_ values are zero. Thus, different from *α*_0_, the substitution of M for Al or N atom in Al_12_N_12_ brings a prominent improvement of first hyperpolarizability (*β*_0_) of the Al_12_N_12_ nanocage, which is because that M-substitution not only destroys the centrosymmetry of Al_12_N_12_ but also makes two kinds of replaced systems possessing the dispersed excess electrons. Two different long-range corrected methods were used to calculate the hyperpolarizability *β*_0_ values, which have been listed in Table 2. Figure 4 manifests the first hyperpolarizability at CAM-B3LYP for M@Al_12_N_11_ and M@Al_11_N_12_ follows the same trend as for BHandHLYP. However, the calculated values at CAM-B3LYP are slightly lower than BHandHLYP.

Additionally, it can be distinctly seen from Table 2 and Figure 4 that the *β*_0_ values of Ca-substitution compounds are greatly larger than those of Be-substitution and Mg-substitution compounds, signifying an evident effect of the M atomic number on the NLO responses of M@Al_12_N_11_ and M@Al_11_N_12_. To be specific, the varying order for *β*_0_ is 861au (Be) < 1979au (Mg) < 6140au (Ca) in the M@Al_12_N_11_ series, which is consistent with the change sequence of *α*_0_ values. That is, the compounds with higher polarizabilities also present relatively higher NLO responses because of the more diffuse excess electrons in them.

The comparison of *β*_0_ values between our proposed excess electron compounds and previously reported alkali-metal substituted compounds is meaningful. In the M@Al_12_N_11_ series, it can be seen that reported alkali-metal substituted compounds exhibit much larger *β*_0_ values of (2,500-9,100) au (in CAM-B3LYP/6-311 + g* level) (Maria et al., 2016) than those (861-6,140) au of our proposed alkaline-earth metal substituted excess electron compounds. However, the resultant comparison of *β*_0_ values is inverse in the M@Al_11_N_12_ series, namely, *β*_0_ values (1,628–3,687) au of alkaline-earth metal-substituted compounds are larger than those (420–770) kcal/mol of alkali-metal-substituted compounds. Thus, when a N atom is substituted by alkaline-earth metal atoms in an Al_12_N_12_ nanocage, excess electron compounds M@Al_11_N_12_ (M = Be, Mg, and Ca) can exhibit larger NLO responses than M@Al_11_N_12_ (M = Li, Na, and K).

**FIGURE 5:**
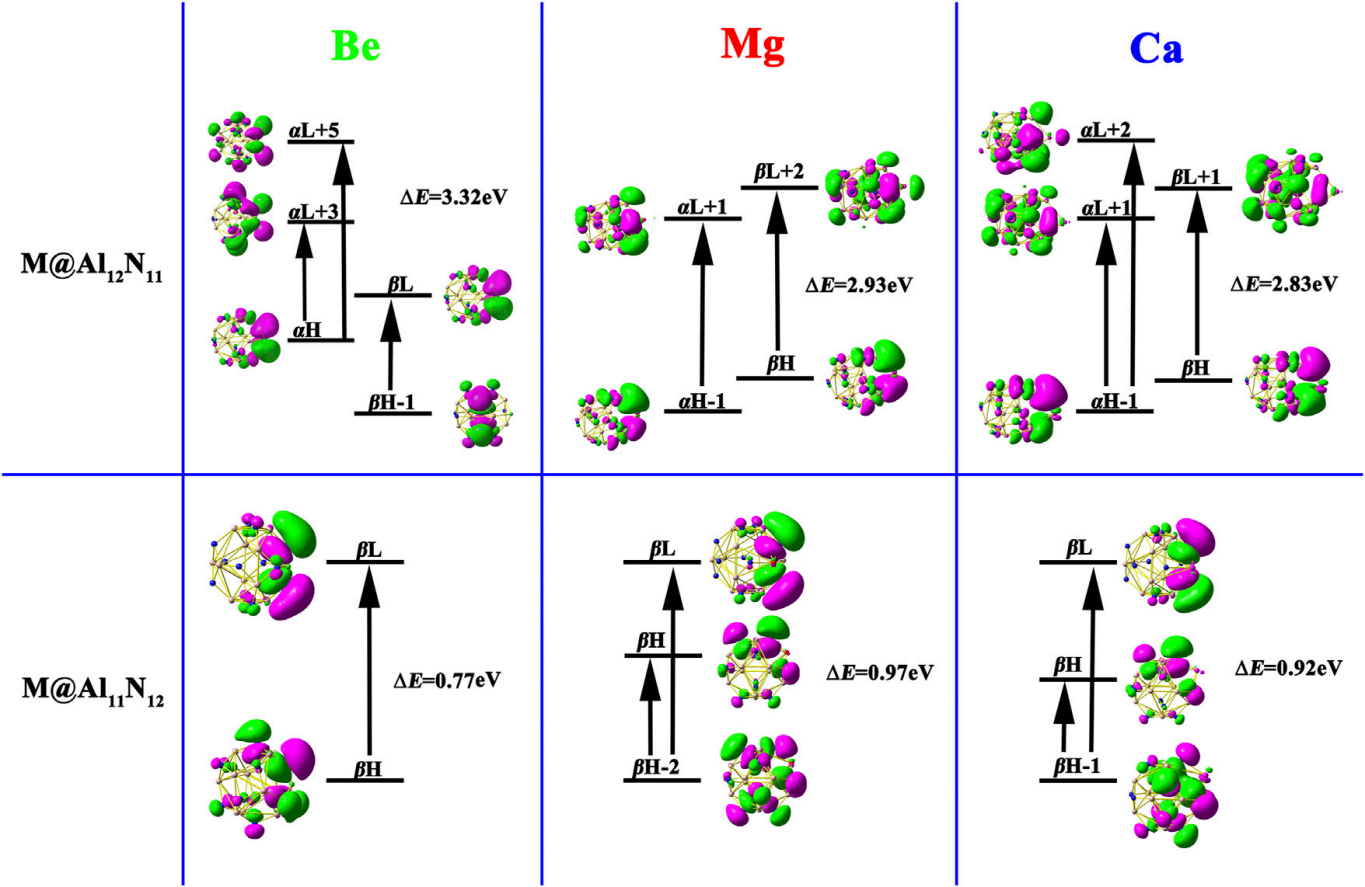
Crucial transition states of M@Al_12_N_11_ and M@Al_11_N_12_ (M = Be, Mg, and Ca) compounds.

For intensive discernment of this significant increase in first hyperpolarizability due to substitution of alkaline-earth metal in the Al_12_N_12_ nanocage, let us consider the simplest two-level model (Maroulis, 1996; Xu et al., 2009), which is derived from the sum-over states method:

$$\beta_{0}\approx \frac{\Delta \mu f_{0}}{\Delta E^{3}},$$ (5)

where involved Δ*E*, *f*_0_, and Δ*μ* are the transition energy, oscillator strength, and difference of the dipole moment between the ground state and the crucial excited state, respectively. The expression distinctly displays that *β*_0_ is inversely proportional to the third power of ∆*E*. Therefore, crucial transition energy plays an important role in the evaluation of *β*_0_. In the current work, the TD-CAM-B3LYP calculations are performed to obtain the dominated excited states of these studied compounds. The crucial transitions and the corresponding Δ*E*, *f*_0_, and Δ*μ* values are presented in Table 2 and Figure 5, respectively. It is noted that the electrons involved in the crucial excited states of these considered species primarily come from their HOMO, HOMO-1, and HOMO-2 orbitals. Meanwhile, from Figure 5, it can be seen that crucial transitions make the electrons more diffuse, which may lead to the large *β*_0_.H

Moreover, compared to the large Δ*E* value (4.759 eV) in pure Al_12_N_12_, the M@Al_12_N_11_ and M@Al_11_N_12_ compounds exhibit much smaller Δ*E* values of (0.77-3.32) eV, which are comparable to those of (1.295–1.982) eV for the alkali metal-based electrides, including Li@(calix [4]pyrrole) (Chen et al., 2005), Li_*n*_-H-(CF_2_-CH_2_)_3_-H (*n* = 1, 2) (Xu et al., 2007), H_4_C_4_N_2_⋯Na_2_ (Ma et al., 2008), and Li@B_10_H_14_ (Muhammad et al., 2009), and far less than (4.6–6.7) eV of M@Al_12_N_11_ and M@Al_11_N_12_(M = Li, Na, and K) (Maria et al., 2016), as well as (1.80–4.76) eV of AEM@Al_12_N_12_ (Ullah et al., 2020). Hence, these small Δ*E* values bring forth the large *β*_0_ values of these proposed alkaline-earth metal-based excess electron compounds.

**FIGURE 6:**
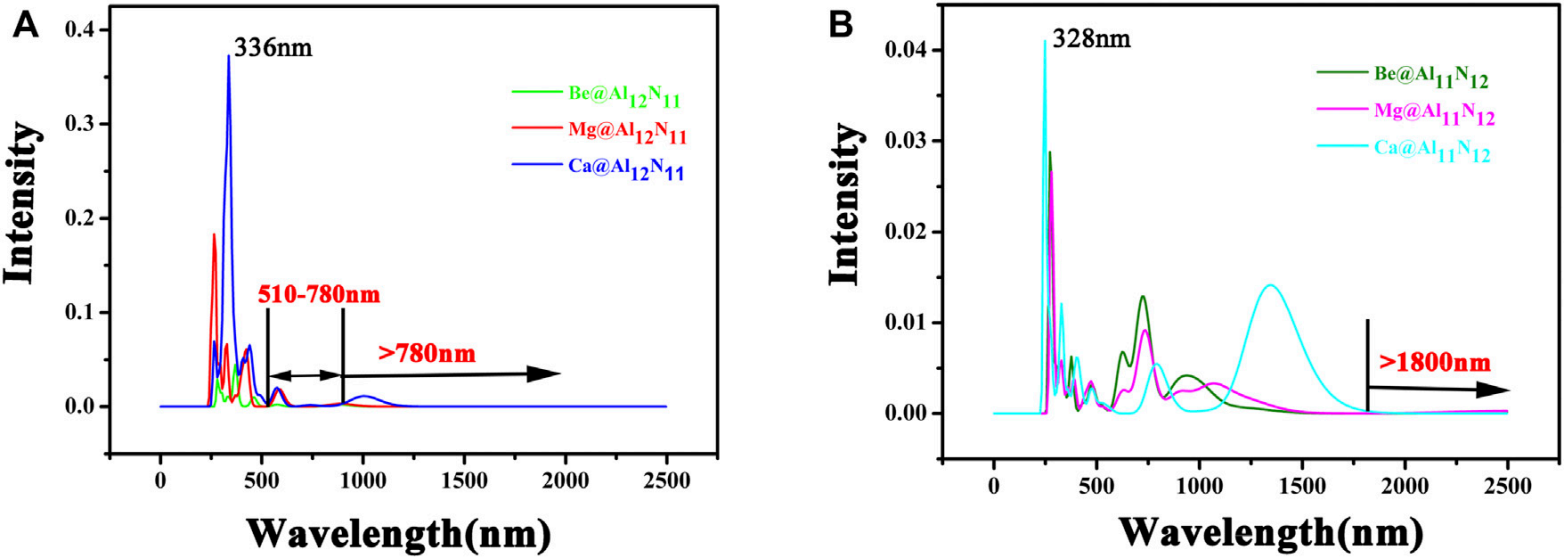
Electronic spectra of M@Al_12_N_11_ and M@Al_11_N_12_ (M = Be, Mg, and Ca) compounds.

It is well-known that the main applications for NLO materials are in doubling frequency and second harmonic generation (SHG). Accordingly, the superior NLO materials not only need large NLO response but also must be transparent under the applied laser region. Therefore, the ultraviolet-visible–infrared (UV-VIS-NIR) absorption spectra of these M@Al_12_N_11_ and M@Al_11_N_12_ compounds are gained and shown in Figure 6. From Figure 6A, it can be seen that the main absorption region of M@Al_12_N_11_ compounds is from 300 to 500 nm. The absorption of these compounds in the infrared spectral region is weak, especially for Be@Al_12_N_11_, there is no absorption in the visible region of (510–780) nm and the infrared spectral region, which suggests that Be@Al_12_N_11_ has satisfying transparency in both the visible region of (510–780) nm and infrared spectral region. In addition, Figure 6B shows that the M@Al_11_N_12_ series have an infrared (IR) transparent region at wavelength >1800 nm. Thus, it is hoped that these six excess electron compounds could be used as new IR NLO materials. Simultaneously, it is observed that they also have an ultraviolet (UV) transparent region at wavelength ˂ 250 nm. Then, they may be taken as a new candidate for UV NLO materials.

## Conclusions

Using the density functional theory (DFT), two new series of excess electron compounds, i.e., M@Al_12_N_11_ and M@Al_11_N_12_ (M = Be, Mg, and Ca), have been obtained and studied theoretically in this work. The substituted effect of alkaline-earth metal on the geometric structures and electronic properties of aluminum nitride (Al_12_N_12_) nanocage has been investigated in detail. Binding energy calculations display that these proposed compounds, particularly the Al-replaced nanocages have high structural stability. In addition, the substitution of alkaline-earth metal for Al and N in Al_12_N_12_ significantly reduces its HOMO–LUMO gap and VIE value, which may bring forth large optical polarizability. More importantly, these studied compounds contain diffuse excess electrons and thus show high NLO responses. Particularly, our results reveal that all these considered compounds show satisfying infrared (IR) transparent region (>1800 nm) and ultraviolet (UV) region (< 250 nm). Thus, we hope that this study could not only provide new candidates of potential NLO molecules but also promote future applications of Al-N fullerene-like nanocages in the field of nonlinear optics.

## Data Availability

The raw data supporting the conclusion of this article will be made available by the authors, without undue reservation.
